# Comparison of Diabetes Management Status between Cancer Survivors and the General Population: Results from a Korean Population-Based Survey

**DOI:** 10.1371/journal.pone.0110412

**Published:** 2014-10-14

**Authors:** Ji-Yeon Shin, Hye Young Shim, Jae Kwan Jun

**Affiliations:** 1 Department of Preventive Medicine, School of Medicine, Eulji University, Daejeon, Republic of Korea; 2 National Cancer Control Institute, National Cancer Center, Goyang, Republic of Korea; Heinrich-Heine University, Faculty of Medicine, Germany

## Abstract

**Purpose:**

This study aimed to determine and compare the prevalences of diabetes awareness, treatment, and adequate glycemic control among cancer survivors in a Korean population and two non-cancer control groups, comprising individuals without a history of cancer but with other chronic diseases (non-cancer, chronic disease controls) and individuals without a history of cancer or any other chronic disease (non-cancer, non-chronic disease controls).

**Methods:**

We analyzed data from 2,660 subjects with prevalent diabetes (aged ≥30 years), who had participated in the 2007–2011 Korea National Health and Nutrition Examination Survey. Awareness was defined as a subject having been diagnosed with diabetes by a clinician. Treatment was defined as a subject who was taking anti-diabetic medicine. Adequate glycemic control was defined as a hemoglobin A1_c_ level of <7%. Multivariable logistic regression and predictive margins were used to evaluate whether awareness, treatment, or adequate glycemic control differed among cancer survivors and the two non-cancer control groups.

**Results:**

Cancer survivors had greater awareness compared with the non-cancer, chronic disease and non-cancer, non-chronic disease control groups (85.1%, 80.4%, and 60.4%, respectively). Although the prevalences of treatment and adequate glycemic control were higher for survivors compared with the non-cancer, non-chronic disease controls, they were lower compared with the non-cancer, chronic disease controls. The prevalence of diabetes treatment was 67.5% for cancer survivors, 69.5% for non-cancer, chronic disease controls, and 46.7% for non-cancer, non-chronic disease controls; the prevalences of adequate glycemic control in these three groups were 31.7%, 34.6%, and 17.8%, respectively.

**Conclusions:**

Cancer survivors were less likely than the non-cancer chronic disease subjects to receive diabetes management and to achieve adequate glycemic targets. Special attention and education are required to ensure that this population receives optimal diabetes care, and the systematic roles for primary care and specialist physicians need to be determined.

## Introduction

Due to advances in early detection and improvement in the treatment of cancer, cancer survivors represent a growing population. There are currently more than 13 million cancer survivors in the U.S., and the long-term cancer survivor population continues to grow [1]. In Korea, the 5-year relative survival rate from 2006–2011 was 64.1%, which represents a 22.9% increase from the 1993–1995 survival rate [2]. In 2011, there were approximately one million cancer survivors estimated in Korea [2].

As the number and survival time of cancer survivors increase, the comorbid conditions and resulting non-cancer mortality of this population have become an important focus of attention. Diabetes is one chronic disease that can strongly influence the prognosis of cancer survivors [3]–[7]. Cancer patients with diabetes showed a 41% higher all-cause mortality compared with individuals without diabetes, according to a pooled-analysis comprising 23 studies on different cancer types [7]. The association of diabetes with cardiovascular mortality is well known [8], and cardiovascular disease is the most important cause of non-cancer deaths in cancer survivors, accounting for 50% of those in the U.S. [9] and for 31.4% in South Korea [10]. Furthermore, diabetes has been reported to increase cancer-specific mortality [3], [4]. Patients with a fasting serum glucose level above 140 mg/dL had a 29% higher death rate from all cancers combined compared with those with <90 mg/dL, [4] and significant associations were found for cancer mortalities of the colon, pancreas, and liver [3], [4]. Diabetes can affect the quality of life (QOL) of cancer survivors. Individuals with both diabetes and cancer had a clinically important and significantly lower health-related QOL (HRQL) than did those with either condition alone [11]. Moreover, second primary cancers are more likely to develop in cancer survivors with diabetes, since hyperglycemia and/or diabetes is associated with an increased risk of cancer [4], [12], [13].

Some cancer survivors, especially those with former childhood, testicular, and hematological cancers, were reported to have an increased risk of developing diabetes or metabolic syndrome, especially considering that chemotherapy and hormonal therapy are known to affect endocrine function [14], [15]. Therefore, continued diabetes monitoring and management are important issues for cancer survivors. However, diabetes management status, such as adherence to treatment or the glycemic control rate, in cancer survivors has not been investigated extensively. Previous studies showed that cancer survivors were less likely to receive recommended care, such as diabetic and preventive care and appropriate follow-ups for heart failure, compared with general population [16], [17]. Thus, overall diabetes management in these individuals is likely to be suboptimal. In this study, we investigated overall diabetes management status, including diabetes awareness, treatment, and adequate glycemic control rates, in cancer survivors compared with two non-cancer control groups; i.e., subjects with no history of cancer but with other chronic diseases, and subjects with no history of cancer or other chronic diseases.

## Materials and Methods

The data were derived from the 2007–2011 Korea National Health and Nutrition Examination Survey (KNHANES). The KNHANES is an ongoing, multicomponent, nationally representative survey of the non-institutionalized Korean population, administered by the Korea Centers for Disease Control and Prevention (KCDC). The survey uses a stratified, multistage probability sampling design, with selections made from sampling units, using household registries, based on geographical area, sex, and age-group. The target population of the survey consisted of non-institutionalized South Korean civilians aged 1 year or older. In 2007 the KNHANES became a year-round investigation employing a rolling sample design. For the KNHANES IV (2007–2009) dataset, 200 sampling units were selected randomly from the primary sampling units encompassing the target population in South Korea. Subsequently, 23 households per sampling unit were subsequently selected (total = 4600 households) in each year. For the KNHANES 2010–2011 dataset, 192 sampling units were selected randomly from the primary sampling units; 20 households per sampling unit were selected (total = 3840 households) in each year [18], [19]. KNHANES consists of a health interview, health examination, and nutritional survey. We used data from the health interview and health examination to obtain information regarding sociodemographic characteristics, medical history, and anthropometric and laboratory measurements. The KNHANES was approved by the KCDC Institutional Review Board, and all subjects signed written informed consent forms. Details of the survey have been described elsewhere [18].

**Figure 1:**
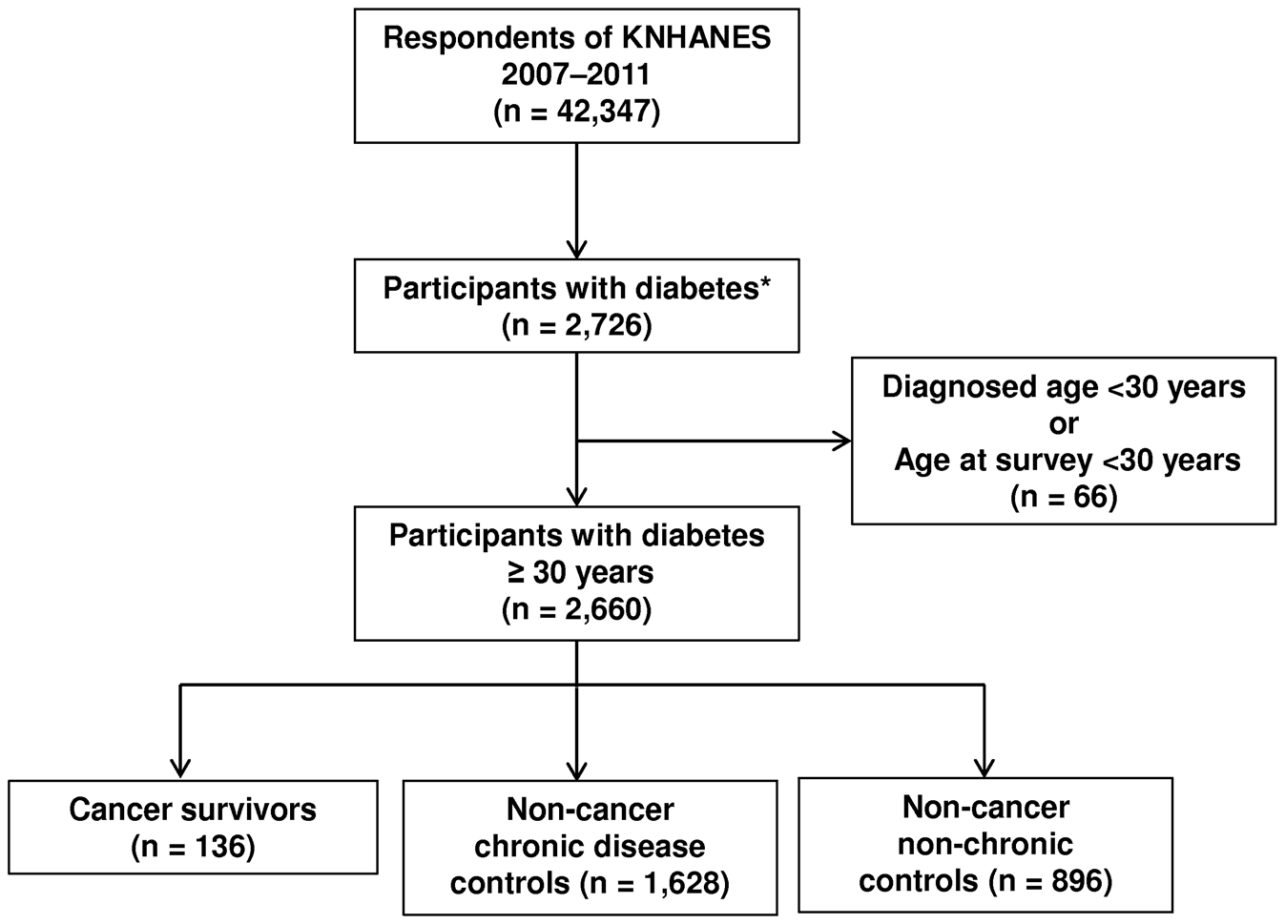
The process used to select the study population. KNHANES, Korea National Health and Nutrition Examination Survey. *Subjects with fasting plasma glucose levels of ≥126 mg/dL, or with a previous clinical diagnosis of diabetes made by a physician, or taking insulin or oral anti-diabetic medication.

We combined data from the 2007–2011 surveys into a large cross-sectional dataset. A total of 4,594 of 6,455 subjects (71.2%) in 2007, 9,744 of 12,528 subjects (77.8%) in 2008, 10,533 of 12,722 subjects (82.8%) in 2009, 8,958 of 10,938 subjects (81.9%) in 2010, and 8,518 of 10,589 subjects (80.4%) in 2011 participated in each survey. Among the 42,347 total subjects, our study sample consisted of the 2,726 subjects with prevalent diabetes. “Prevalent diabetes” was applicable to individuals with a fasting plasma glucose level of ≥126 mg/dL or to subjects responding “yes” to any of the following questions: “Have you ever been told by a doctor that you have diabetes?”; “Are you now taking insulin?”; and “Are you currently taking oral hypoglycemic agents?” To limit the analysis to adults with type 2 diabetes, we excluded subjects whose age at diagnosis was <30 years [20] or whose age at the time of the survey was <30 years. After applying the exclusions, 2,660 subjects remained for analysis (Figure 1).

Among the prevalent diabetes subjects, those who had been diagnosed with diabetes by a clinician were considered to be aware of their diabetes status. Subjects who were on pharmacological treatment (either insulin, oral anti-diabetic drugs, or both) for diabetes were considered as “treated”. Subjects were characterized as displaying adequate glucose control when their hemoglobin A1_c_ (HbA1c) levels were <7% [21]. We classified the subjects as cancer survivors if they answered “yes” to the interview question, “Have you ever been told by a doctor that you have cancer or a malignancy of any kind?” The type of cancer and the age at diagnosis were also noted. Since we assumed that frequent contacts with health professionals were associated with the quality of care the patient received, we separated the remaining subjects into two non-cancer control groups: those without cancer but with chronic disease and those without cancer or any other chronic disease. In accordance with a previous study, we operationally defined chronic disease as incurable and usually associated with a life-time increase of medical encounters [22]. Participants who had been diagnosed by a physician, with hypertension, arthritis, heart disease, stroke, asthma, or chronic obstructive pulmonary disease, comprised the chronic disease group [22].

Blood samples were obtained from subjects after a minimum 8-h fast. The serum fasting glucose levels were measured enzymatically at a central laboratory, using Advia 1650/2400 (Siemens, New York, USA) during the 2007 KNHANES and using an automatic analyzer 7600 (Hitachi, Tokyo, Japan) during the 2008–2011 KNHANES. In the 2007–2010 KNHANES, HbA1c was measured in the subjects with fasting plasma glucose levels ≥126 mg/dL or in those who were taking insulin or oral anti-diabetic medication, while in the 2011 KNHANES, HbA1c was measured in all subjects 10 years of age or older. HbA1c levels were measured using a high-performance liquid chromatography Varian II assay (Bio-Rad, Carlsbad, CA, USA) in the 2007 KNHANES and using a high-performance liquid chromatography-723G7 (Tosoh, Japan) in the 2008 KNHANES. Rigorous quality-control (QC) programs were employed in both surveys, and HbA1c measurements were reportedly comparable [23]. The detailed methods for comparing and assessing the validity and reliability of each survey are described elsewhere [23], [24].

All statistical analyses were performed using the software package SAS (ver. 9.2, SAS Institute, Cary, NC, USA), with a survey procedure that adjusted for the complex survey design and included appropriate sampling weights to obtain accurate estimates representative of the non-institutionalized Korean population (according to KCDC guidelines) [18]. A descriptive analysis was used to assess the characteristics of the cancer survivors and control subjects with diabetes. A chi-square analysis was used to compare the categorical variables among the three groups. Next, we compared the prevalence of diabetes awareness, treatment, and adequate glycemic control among the three groups. We used multivariable logistic regression analyses to compute adjusted prevalence estimates (*i*.*e*., predicted population margins) as well as adjusted odds ratios (ORs) and confidence intervals (CIs) [25], [26]. We considered age (30–49, 50–59, 60–69, and ≥70 years), sex, education (elementary school or less, middle or high school graduates, and college or higher graduates), and body mass index (BMI) as potential confounders when calculating the adjusted prevalence, according to previous studies [20], [27], [28]. BMI was calculated as weight in kilograms divided by height in meters squared. The statistical significance of the differences in prevalence was determined using the *t* statistic derived from the general linear contrast procedure. We adjusted for multiple tests by applying the Bonferroni correction [26]. Since glycemic control is associated with diabetes duration [29], we conducted additional subgroup analyses, for treatment and adequate glycemic control, according to diabetes duration (*i*.e., ≤5 years and >5 years). This procedure is in line with previous studies [30], [31]. In the HbA1c-based analyses of glycemic control, we excluded subjects whose HbA1c levels had not been measured; therefore, a total of 2,556 subjects were included in the analyses. We considered a two-tailed *p* value<0.05 as statistically significant.

## Results

Our study population comprised 136 cancer survivors, 1,628 non-cancer chronic disease controls, and 896 non-cancer non-chronic disease controls, all with diabetes, and their mean ages were 65.2, 62.6, and 52.8 years, respectively. Of the 136 cancer survivors, 58.8% reported having one of the following four types of cancer: stomach (n = 26), colorectal (n = 21), cervical (n = 20), or breast cancer (n = 13).

Table 1 presents the descriptive analyses for the cancer survivors and the two control groups. Cancer survivors had somewhat different sociodemographic characteristics compared with the non-cancer non-chronic disease controls. The cancer survivors were more likely to be older, female, unmarried, to have a lower level of education and a lower monthly income. Moreover, they were more likely to have had a longer diabetes duration. In contrast, cancer survivors and non-cancer chronic disease controls had similar sociodemographic characteristics, with the exceptions that the former were more likely to be older, non-obese, and to have-longer-term diabetes than the latter.

**Table 1:** Characteristics of cancer survivors and controls with prevalent diabetes among the pooled subjects from the 2007–2011 Korea National Health and Nutrition Examination Survey.

				All		Cancer survivors (n = 136)		Non-cancer chronic disease controls (n = 1,628)		Non-cancer non-chronic disease controls (n = 896)		
Variable				Unweighted No.	% (SE)	%	(SE)	%	(SE)	%	(SE)	*P* value
Age (years)												
	30–49			425	24.4(1.2)	12.4	(3.5)	12.0	(1.2)	43.2	(2.1)	<0.001
	50–59			593	28.2(1.1)	11.9	(3.1)	27.7	(1.4)	30.5	(1.8)	
	60–69			881	25.9(0.9)	41.4	(4.5)	31.4	(1.3)	16.7	(1.3)	
	≥70			761	21.5(0.9)	34.2	(3.8)	29.0	(1.3)	9.7	(0.9)	
Gender												
	Male			1333	55.6(1.1)	43.5	(4.5)	49.1	(1.5)	66.1	(1.9)	<0.001
	Female			1327	44.4(1.1)	56.5	(4.5)	50.9	(1.5)	33.9	(1.9)	
Education^a^												
	Middle school and under			1306	42(1.2)	53.7	(4.5)	52.9	(1.5)	25.2	(1.6)	<0.001
	Middle-high school			1018	42.9(1.2)	36.3	(4.4)	37.7	(1.4)	51.0	(1.9)	
	College and above			324	15.1(1)	10.0	(3.6)	9.4	(1.0)	23.7	(1.8)	
Marital status												
	Married			2026	77.7(1.0)	69.0	(4.5)	72.9	(1.4)	85.4	(1.3)	<0.001
	Unmarried (Single, divorced, widowed)			634	22.3(1.0)	31.0	(4.5)	27.1	(1.4)	14.6	(1.3)	
Monthly household income												
	<1000 USD			925	27.8(1.1)	34.4	(4.5)	34.6	(1.5)	17.4	(1.4)	<0.001
	1000–3000 USD			944	36.9(1.2)	28.8	(4.2)	35.1	(1.5)	40.2	(2.1)	
	≥3000 USD			791	35.3(1.3)	36.8	(4.2)	30.2	(1.6)	42.4	(2.1)	
Health insurance type												
		National Health Insurance		2469	93.5(0.6)	92.1	(2.6)	92.6	(0.8)	94.9	(0.9)	0.11
		Medicaid/none/others		191	6.5(0.6)	7.9	(2.6)	7.4	(0.8)	5.1	(0.9)	
Residential area												
		City		1901	76.8(1.8)	74.0	(3.7)	76.0	(1.9)	78.2	(2.2)	0.43
		Rural		759	23.2(1.8)	26.0	(3.7)	24.0	(1.9)	21.8	(2.2)	
Duration of diabetes^a^ (years)												
		≤1		312	16.9(1.1)	16.4	(3.7)	15.3	(1.3)	20.0	(2.1)	<0.001
		2–5		667	34.6(1.3)	21.0	(4.4)	34.6	(1.6)	36.4	(2.5)	
		>5		1053	48.5(1.4)	62.6	(4.8)	50.1	(1.7)	43.6	(2.6)	
Body mass index (kg/m^2^)												
			<25.0	1387	50.8(1.2)	58.4	(4.5)	45.1	(1.6)	58.2	(1.9)	<0.001
			25.0–29.9	1091	41.7(1.2)	36.3	(4.3)	46.6	(1.6)	35.1	(1.8)	
			≥30	182	7.5(0.7)	5.3	(1.8)	8.3	(0.9)	6.7	(1.1)	

a The total number of subjects among these categories does not equal 2,660 due to missing data.

Table 2 lists the prevalences, odds ratios (ORs), and 95% confidence intervals (CIs) for the diabetes awareness, treatment, and adequate glycemic control variables of each group. Cancer survivors had a higher awareness than did both the non-cancer chronic disease controls and non-cancer non-chronic disease controls (85.1%, 80.4%, and 60.4%, respectively). Following adjustment for other covariates, awareness remained higher in cancer survivors compared with the control groups. Diabetes awareness ORs (which cannot be interpreted as relative risks) in the non-cancer chronic disease and cancer survivor groups, were 2.0 (95% CI: 1.6, 2.7) and 2.5 (95% CI: 1.4, 4.4), respectively, when the non-cancer, non-chronic disease group was used as the reference (Table 2, Model 2).

**Table 2:** Prevalence, odds ratios (ORs), and 95% confidence intervals (CIs) for diabetes awareness, treatment, and adequate glycemic control in the cancer survivor and control groups with prevalent diabetes.

		Cancer survivors		Non-cancer chronic disease controls		Non-cancer non-chronic disease controls	
		%	OR	%	OR	%	OR
Variables		(95% CI)	(95% CI)	(95% CI)	(95% CI)	(95% CI)	(95% CI)
Awareness							
	Crude	85.1	3.7	80.4^b^	2.7	60.4^a^	Reference
		(76.2–91.0)	(2.0–6.9)	(77.8–82.8)	(2.2–3.4)	(56.4–64.2)	
	Model 1	83.0	2.4	78.8^b^	1.8	67.1^a^	Reference
		(73.8–89.5)	(1.3–4.4)	(75.9–81.4)	(1.4–2.4)	(62.8–71.1)	
	Model 2	81.4	2.5	78.4^b^	2.0	64.0^a^	Reference
		(72.0–88.2)	(1.4–4.4)	(74.6–81.8)	(1.6–2.7)	(58.8–68.9)	
Treatment							
	Crude	67.5	2.4	69.5^b^	2.6	46.7^a^	Reference
		(57.1–76.4)	(1.5–3.8)	(66.7–72.1)	(2.1–3.2)	(42.8–50.6)	
	Model 1	62.8	1.5	66.6^b^	1.8	53.2^c^	Reference
		(52.2–72.2)	(0.9–2.4)	(63.6–69.4)	(1.4–2.2)	(49.0–57.3)	
	Model 2	60.5	1.5	65.0^b^	1.8	51.1^c^	Reference
		(49.4–70.5)	(0.9–2.3)	(60.9–68.9)	(1.4–2.2)	(46.0–56.2)	
Adequate glycemic control							
	Crude	31.7	2.1	34.6^b^	2.4	17.8^a^	Reference
		(23.2–41.6)	(1.3–3.5)	(31.9–37.3)	(1.9–3.1)	(15.0–21.1)	
	Model 1	27.1	1.5	31.3^b^	1.8	20.0^c^	Reference
		(19.1–36.8)	(0.9–2.4)	(28.4–34.4)	(1.4–2.3)	(16.9–23.5)	
	Model 2	25.2	1.5	29.5^b^	1.8	18.7^c^	Reference
		(17.5–34.8)	(0.9–2.4)	(25.6–33.6)	(1.4–2.3)	(15.1–22.8)	

Model 1 was adjusted for age and sex; Model 2 was also adjusted for education and body mass index.

We adjusted for multiple tests by applying the Bonferroni method.

Awareness: proportion of prevalent diabetes subjects who had been diagnosed with diabetes by a clinician.

Treatment: proportion of prevalent diabetes subjects who were receiving pharmacological treatment (either insulin, oral anti-diabetic drugs, or both).

Adequate glycemic control: proportion of prevalent diabetes subjects displaying hemoglobin A1c (HbA1c) levels of <7%.

a *p*-value<0.05 when compared with cancer survivors.

b *p*-value<0.05 when compared with non-cancer non-chronic disease controls.

c *p*-value<0.15 when compared with cancer survivors.

The prevalence of diabetes treatment in the cancer survivors (67.5%) was significantly higher compared with the non-cancer, non-chronic disease controls (46.7%), but lower compared with the non-cancer, chronic disease controls (69.5%). The prevalence of diabetes treatment in the cancer survivor group was lower compared with the non-cancer, chronic disease controls following adjustment for other covariates, but this difference was not statistically significant.

The prevalence of adequate glycemic control was also lower, but not significantly so, in the cancer survivors compared with the non-cancer chronic disease controls (31.7% and 34.6%, respectively). However, the prevalences in both groups were significantly higher compared with the rate of the non-cancer, non-chronic disease control group (17.8%). The non-cancer chronic disease control group had the highest prevalence of adequate glycemic control, followed by the cancer survivor and non-cancer, non-chronic disease control groups, although group differences decreased following adjustment for age, sex, education, and BMI (29.5%, 25.2%, and 18.7%, respectively; Table 2, Model 2).

**Figure 2:**
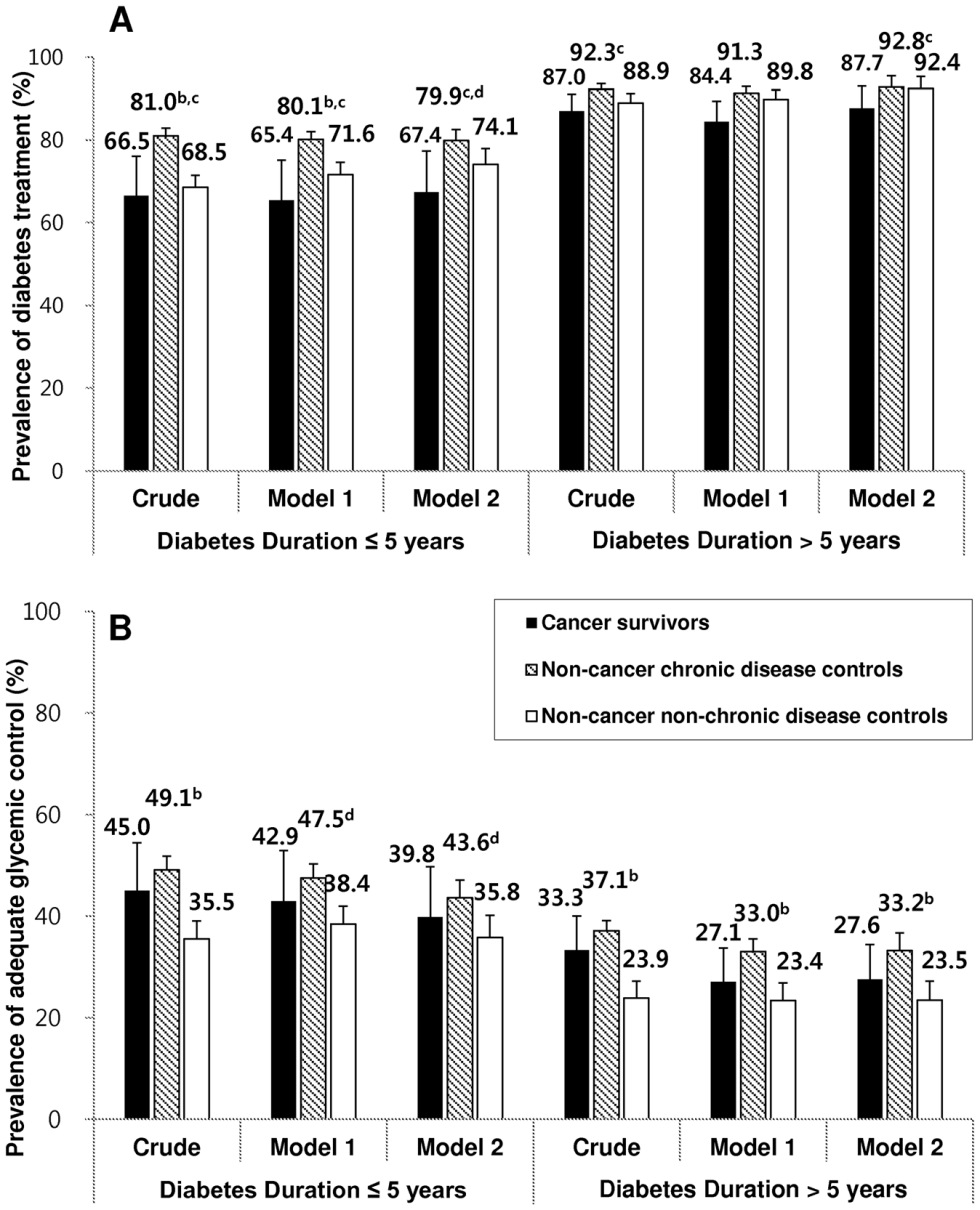
Prevalence of: (A) diabetes treatment; and (B) adequate glycemic control, among the cancer survivor and control groups with prevalent diabetes, according to diabetes duration. Model 1 was adjusted for age and sex; Model 2 was additionally adjusted for education and BMI. We adjusted for multiple tests by applying the Bonferroni method. ^a^ *p*-value<0.05 when compared with cancer survivors. ^b^ *p*-value<0.05 when compared with non-cancer non-chronic disease controls. ^c^ *p*-value<0.15 when compared with cancer survivors. ^d^ *p*-value<0.15 when compared with non-cancer non-chronic disease controls.

Figure 2 illustrates the prevalences of diabetes treatment and adequate glycemic control, following stratification according to diabetes duration (≤5 years and >5 years). Cancer survivors showed the lowest prevalence of treatment among the three groups, regardless of duration. For subjects with a diabetes duration of ≤5 years, the prevalences of adequate glycemic control in the cancer survivors, non-cancer chronic disease controls, and non-cancer, non-chronic disease controls were 66.5%, 81.0%, and 68.5%, respectively: the cancer survivor and non-cancer, chronic disease control group difference was marginally significant. Even though the overall prevalence of treatment in subjects with a diabetes duration of >5 years was higher compared with subjects with a duration of ≤5 years, the trends observed in each model did not differ (Figure 2a).

Moreover, and following stratification according to diabetes duration, the prevalence of adequate glycemic control of the cancer survivor group was lower compared with the non-cancer, chronic disease group, but higher compared with the non-cancer, non-chronic disease control group. However, the differences among the survivors and control groups were not statistically significant. For the ≤5-year diabetes duration subjects, the prevalence of adequate glycemic control was 45.0% in the cancer survivor group, 49.1% in the non-cancer chronic disease control group, and 35.5% in the non-cancer non-chronic disease control group. These values were higher than those of the >5-year diabetes duration subjects (33.3%, 37.1%, and 23.9%, respectively). This trend did not change after adjusting for other covariates (Figure 2b).

## Discussion

To our knowledge, this is the first study to compare the diabetes management status of cancer survivors with the general population based on a nationally representative population. According to the results, diabetes awareness in cancer survivors was significantly higher than in both non-cancer control groups. However, the prevalences of diabetes treatment and adequate glycemic control of cancer survivors were lower compared with that of the non-cancer, chronic disease controls. Even after adjusting for or stratifying by covariates that could affect diabetes management, the results remained the same.

Our finding that cancer survivors demonstrated higher levels of awareness of diabetes and a higher prevalence of adequate glycemic control, compared with non-cancer, non-chronic disease control subjects, appears to be associated with the typically greater number of medical contacts made by the former group. Non-cancer, non-chronic disease subjects might predominately comprise individuals who do not visit their physicians, such that the frequency of undiagnosed diabetes could be higher in this population compared with the other groups. The frequency of medical encounters has been suggested to be an important determinant of the quality of care that patients receive [32]. A previous study reported that breast cancer survivors were more likely to receive routine preventive services than individuals without a history of cancer [32]. In a Korean multicenter study, cancer survivors with hypertension were more likely to regularly take antihypertensive medication than the general population [33]. In addition, cancer survivors were more likely to undergo cancer screening in accordance with guidelines than those without a history of cancer [22], [32], [34]. We found that the non-cancer chronic disease control subjects, who were generally considered to have more frequent contacts with physicians than the non-cancer non-chronic disease controls, also displayed higher awareness than did the non-cancer non-chronic disease subjects; this finding demonstrates the potential impact of frequent medical contact on diabetes awareness.

Interestingly, even though the subjects of both groups were considered to have frequent contact with physicians, the cancer survivors showed lower treatment and control than those in non-cancer chronic disease controls. There are several possibilities for this. First, cancer survivors tend to have greater difficulty maintaining general medical check-ups and receiving comprehensive health management for other diseases than non-cancer chronic disease controls. Even though cancer survivors visit oncology specialists at tertiary hospitals for cancer-related follow-up, it is difficult for the oncology specialist to provide comprehensive care. In fact, oncologists do not always assume the primary care role that cancer patients expect of them [17], and to some extent, they are not willing or equipped to deal with such problems [35]. In addition, cancer patients often expect their oncologist to be their sole care provider [36], potentially losing contact with non-cancer providers who are critical for overall health care [17]. In Korea, there is neither a family doctor referral system, designated physician, nor an organized care system for long-term follow-up of cancer survivors. Thus, the difficulties in management of chronic comorbid disease for cancer survivors appear to be exacerbated in Korea.

Second, cancer survivors might frequently be unaware of the effects of diabetes and poor glycemic control on their disease prognosis, or of the risk that it confers regarding the development of other cancers. Previous studies suggest that cancer survivors are characterized by a knowledge deficit regarding the late effects of treatment and individual health risks [37], [38]. In contrast, patients with non-cancer-related chronic diseases, such as hypertension and cardiovascular disease, are relatively more likely to be aware of the risk that diabetes imposes upon their disease. According to the Health Belief Model, perceived susceptibility is critical for healthy behaviors [39]. In addition, perceived benefits (related to treatment efficacy) and perceived severity contribute to sick-role behavior after diagnosis [39]. If cancer patients realize the impact of diabetes on disease prognosis and take the risk of diabetes more seriously, then their health behaviors are more likely to change.

Third, diabetes treatment and management are not likely major priorities for cancer patients, regardless of whether they recognize the risk of diabetes. The work by Earle and Neville indicated that having a prior cancer diagnosis can shift attention away from other important health issues [17], and Shin *et al*. argued that some cancer survivors are relatively ignorant of their health needs outside of cancer, tending to only focus on cancer treatment [40]. In many cases, diabetes management is a lower priority than cancer care, causing cancer survivors to be negligent in diabetes management.

In regards to the second and third possibilities, educating cancer survivors about the importance of diabetes management may be beneficial. If the patients recognize the impacts of uncontrolled blood sugar on cancer-related prognoses and/or cancer-unrelated health, diabetes care may increase enough in priority to improve their self-glycemic control.

The question as to who is responsible for patient education in our current healthcare system is connected to the question of which system should be applied to the long-term care of cancer survivors. During the last few years, there has been intense discussion in regards to follow-up care of cancer survivors [35]. The American Society of Clinical Oncology recommends promoting successful models of survivor care and tools that optimize the transition process between oncologist and primary care providers to achieve high-quality cancer survivor care [36]. Studies have shown that survivors who visited both primary care physicians (PCP) and oncologists received better comorbidity management and care than did cancer patients who only visited oncologists [16], [17]. Since an oncologist alone cannot be responsible for all areas of care, it is desirable that PCPs take partial responsibility for care of cancer survivors. Yet, this creates ambiguity in several areas; e.g., screening for specific cancers, screening for other cancers, and ongoing comorbidity management [35]. Further studies and discussions are needed to ensure that the roles of oncology specialists and PCPs are systematically coordinated in the future.

This study had a number of limitations. The KNHANES was not designed to investigate cancer survivorship. For this reason, the number of cancer survivors included in the study was relatively small. The limited sample size of the cancer survivor group caused the predictions derived from the subgroup analyses less stable and more difficult to conduct further analyses based on factors such as cancer type and age. Moreover, KNHANES classified cancer into only the six-most prevalent types in Korea (gastric, liver, colorectal, breast, cervical, and lung cancers), and consequently data for the other types were absent, creating several missing values for the “cancer type” variable. In addition, because additional information, such as the cancer stage and treatment method (*i*.*e*., the use of chemotherapy or radiotherapy), was not collected in KNHANES, we could not assess the associations of these variables with diabetes management. Second, cancer and other chronic disease diagnoses and the use of diabetes medication were self-reported, which could lead to misclassification bias. Third, we were unable to consider the effects of order. Due to the numerous missing values among the dates of diagnosis, we could not confirm whether cancer, or other chronic diseases, manifested before or after the diabetes diagnosis. Fourth, we could not rule out the possibility of survival bias, because the KNHANES uses a cross-sectional design. Therefore, our finding that the prevalences of treatment and adequate glycemic control of the cancer survivors and non-cancer chronic disease patients were higher, compared with the non-cancer, non-chronic disease group, could be due to the poor health status of the former subjects, which had either already resulted in death or prevented their participation in the investigation.

Despite these limitations, our finding that cancer survivors had a suboptimal diabetes management status compared to that of the non-cancer chronic disease group suggests there is room for improvement in the management of diabetes in cancer survivors. Taking this into consideration, the additional finding that the prevalences of treatment and adequate glycemic control were lower in cancer survivors compared with the non-cancer, chronic disease group, despite greater awareness of diabetes in the cancer survivors, strongly indicates that there are certain barriers to adequate diabetes management in cancer survivors. Our findings illustrate the issues with the health care system for cancer survivors in Korea, in terms of the ambiguity in care responsibility between oncologists and PCPs. Inadequate glycemic control in cancer survivors could lead to severe cardiovascular or oncogenic consequences, and special attention to diabetes management and proactive education is needed to ensure that survivors receive optimal diabetes care. Regardless of who is responsible, general primary care issues, including glycemic control in cancer survivors, should be addressed with greater priority. Further studies in this area are warranted to determine the optimum roles of primary care and specialist physicians.
